# Measuring what matters to the patient: health related quality of life after aortic valve and thoracic aortic surgery

**DOI:** 10.1007/s11748-017-0830-9

**Published:** 2017-09-13

**Authors:** Frederiek de Heer, Arjen L. Gökalp, Jolanda Kluin, Johanna J. M. Takkenberg

**Affiliations:** 1 Dept. of Cardio-Thoracic Surgery, Academic Medical Center, P.O. Box 22660, 1105 AZ Amsterdam, The Netherlands; 2 Dept. of Cardio-Thoracic Surgery, Bd563, Erasmus University Medical Center, P.O. Box 2040, 3000 CA Rotterdam, The Netherlands

**Keywords:** Health-related quality of life, Thoracic aortic surgery, Aortic valve replacement

## Abstract

With improved outcomes following cardiac surgery, health related quality of life (HRQoL) gains increasing importance for the better judgement of choosing the preferred treatment strategy in the individual patient. The physician perception of patient preferences can differ considerably from actual patient preferences, underlining the importance of gathering evidence of actual patient preferences before and quality of life after cardiac surgery. The objective of the current review is to provide an overview of current insights into the quality of life measurements after aortic valve and thoracic aortic surgery and to provide starting points for the application of HRQoL measurements toward the future. The amount and level of evidence on HRQoL outcomes after aortic valve and thoracic aortic surgery seems to be insufficient. Little has been investigated about the natural course of HRQoL after cardiac surgery, HRQoL outcomes between different surgical strategies, HRQoL outcomes between surgical patients and the general population, the different factors influencing HRQoL after cardiac surgery, and the effect of HRQoL on healthcare costs. More prospective studies should be performed, taking into account the knowledge gaps that need to be filled. Computerized adaptive testing methods through open source programs can be implemented to keep the burden to the patient as low as possible and catalyze the use of these tools. Our cardiovascular surgery community has the responsibility to deliberate how it can proceed to effectively fill in these knowledge gaps, and use this newfound knowledge to improve shared treatment decision making, patient outcomes, and ultimately optimize health care efficiency.

## Introduction

Traditionally, long-term outcomes after cardiac surgery have been reported in terms of mortality, morbidity, and the recurrence of disease [1]. With improved outcomes following cardiac surgery, health related quality of life (HRQoL) gains increasing importance for the better judgement of choosing the preferred treatment strategy in the individual patient. This specifically applies for the field of elective surgery on the thoracic aorta when asymptomatic patients are operated on prognostic grounds. The literature gives a wide view of the variety of HRQoL questionnaires which has been used and—surprisingly—are still not widely adapted in prognostic research. The objective of the current review is to provide an overview of current insights into quality of life measurements after aortic valve and thoracic aortic surgery in adult patients and to provide starting points for the application of HRQoL measurements toward the future.

## Why it is important to measure HRQoL

Professional medical values and ethics have evolved since Hippocrates. Nevertheless, the core values are unscathed: the first concern of physicians is the care of their patients. It is the duty of physicians to listen to their patients, to respect their views and give them information in a way they can understand [2]. In the current era, where often multiple treatment modalities are available, a physician wants to deliver the best care for his or her individual patient, given co-morbidities, treatment options, outcomes, societal costs and patient preferences. Quality of life as an outcome measure adds an important dimension to clinical outcomes, allowing the introduction of the concept of quality adjusted life years (QALY). But how accurate is physician perception of patient preferences and how can we best assess quality of life? In the domain of cardiovascular surgery and colorectal cancer it was demonstrated that the physician perception of patient preferences can differ considerably from actual patient preferences [3]. It underlines the importance of gathering evidence of actual patient preferences before and quality of life after cardiac surgery. From a patient perspective it is worth knowing how long it takes to recover from surgery, and if and when one can go back to living their life to the fullest. Surgeons can analyze aortic root diameters, reoperation rates and survival, but measuring a person’s emotional state or physical impairment is more challenging. Tailoring the optimal treatment strategy to the individual patient can be particularly delicate in heart valve and aortic root surgery, as the available treatment options carry different value-sensitive advantages and disadvantages such as bleeding and thrombo-embolic risks, reoperations risks, valve sound and strict anticoagulation management. There is emerging evidence that patients requiring heart valve replacement want to be involved more in their treatment decision making and that involved patients are better informed, less anxious and depressed, and have a better mental well-being [4]. This is not only important for the patient: from a societal perspective it is crucial that costs of healthcare are contained, while achieving better outcomes. Patient empowerment—including addressing values, preferences and quality of life—is thought to be one of the cornerstones of health care cost containment. Bradley et al. nicely describe the main aspects of ‘value measurements’ in the field of cardiovascular care [5]. Healthcare value is defined as ‘health outcomes achieved relative to the costs of care’ and they underline the definition proposed by porter that outcomes should reflect the ‘health circumstances most relevant to patients’ [6].

For all the reasons mentioned above it is crucial that in the cardiovascular domain, with complex patients undergoing complex high-tech procedures, we put emphasis on measuring HRQoL in our outcomes research, and apply this knowledge to further improve our clinical practice.

## How to measure HRQoL after cardiac surgery

Four decades ago one of the first reports on HRQoL after cardiac surgery was described by Ross et al. [7] Their survey showed an overall improvement in the quality of life for the majority of patients having open-heart surgery at a time that the field of cardiac surgery had passed beyond the development phase into a standard practice and had gained wider acceptance. There are many ways to measure HRQoL. In the field of thoracic aortic surgery recently Jarral et al. conducted a systematic review that revealed a total of 17 different HRQoL instruments. The most commonly used generic HRQoL instrument was the Short Form 36-Item Health Survey (SF-36) (67% of the studies). The other instruments that were used in more than two studies were: hospital anxiety and depression score (HADS), RAND SF-36, Karnofsky activity scale, SF-12, illness intrusiveness rating scale, sickness impact profile questionnaire and a ‘General Health perception questionnaire’. EuroQOL-5D (EQ-5D), a preference-based measure, was found in two studies. Even valve-specific measures were not used frequently. For example, the valve disturbance questionnaire, was used once [8]. Additionally, worth mentioning is a survey which combines HRQoL questions with functional measurements, like the Duke Activity Status Index (DASI) [9]. Below a short description of the most frequently used instruments is given:

### SF-36

The most frequently used instrument is SF-36: it is a multipurpose, short-form with 36 questions. It yields an eight-scale profile of scores as well as physical and mental health summary measures. It is a generic measure, as opposed to one that targets a specific age, disease, or treatment group [10]. Although it has a generic approach, SF-36 is the most widely used survey in thoracic aortic surgery. In 1996 the SF-36 is updated in version 2. Modifications include format changes, wording revisions, and changes in the number of response choices, which resulted in greater readability, reliability, and validity [11]. Moreover, the interpretation of SF-36v2 results has been greatly simplified with the norm based scoring of its health domain scales and component summary measures. Instead of a scale from 0 to 100 the norm based scales refer to the deviation from the general population norm as these values are built into the scoring algorithm. An individual respondent score above 45 and group mean scores above 47 can be interpreted as being above the average range for the general US population. Norm scores are also being developed for specific age groups or countries outside the US [12].

#### Hospital anxiety and depression score (HADS)

Hospital anxiety and depression score is developed as a clinical screening tool to assess anxiety and depression among patients in hospitals. The instrument comprises 14 questions and results in two sum scores: one for anxiety and one for depression. Higher scores indicate more change to have a mood disorder. The HADS is a well-accepted, valid, and reliable screening tool [13].

#### HRQoL survey correlated with functional measurements

An example of a functional assessment based on activities of daily living and cardiovascular fitness, assessed using a self-administered questionnaire is the Duke Activity Status Index (DASI). Peak oxygen uptake was considered the gold standard for cardiovascular functional capacity. DASI was developed by taking into account the empirical correlations of questionnaire items with peak oxygen uptake, clinical judgment and information from previous studies about the activities best representing different aspects of physical functioning [9].

#### Valve specific questionnaire

The valve-specific questionnaire determines the effects of valve related items such as bothersome sounds, frequency of medical visits, and fear of potential complications such as bleeding or reoperation for valve failure. It includes the powerful question “If I had to do it over again, would I make the same decision to have surgery?”, which covers a weighing of all pro’s and con’s [14].

## What is known?

Several studies have studied the effect of cardiac surgery on HRQoL. Chocron et al. [1] compared HRQoL before and after open heart surgery using the Nottingham Health Profile. They found that HRQoL scores improved significantly in an average of 80% of patients and 91% of patients reported the operation improved their lives. However, these outcomes apply to the general cardiac patient population, and not specific cardiac surgery subgroups. HRQoL scores, like any other outcome measure, may differ between the different cardiac surgery procedures. Therefore, we reviewed the literature on HRQoL focusing on aortic valve surgery and thoracic aortic surgery.

### Aortic valve surgery

#### Mechanical valves versus tissue valves

Two studies investigating HRQoL using the SF-36, found that patients receiving a mechanical or tissue aortic valve prosthesis report comparable quality of life after their operation [15, 16]. Another two studies investigating HRQoL using the SF-36, found that on average tissue valve recipients and aortic valve repair patients report better quality of life than mechanical valve recipients [17, 18]. The studies comparing quality of life after surgery with the general population using the SF-36 found no significant differences [16, 19]. No associations were found between the quality of life scores and preoperative NYHA scores or event rates [17, 19].

#### Aortic valve repair versus aortic valve replacement

A landmark study by Aicher et al. [20] compared quality of life after aortic valve repair and aortic valve replacement using mechanical valves and pulmonary autografts, using the SF-36, Hospital Anxiety and Depression Scale (HADS), Cardiac Anxiety Questionnaire (CAQ), and valve-specific questions. SF-36 outcomes in most domains were comparable between aortic valve repair and pulmonary autograft patients, but significantly better in aortic valve repair compared to mechanical valve replacement. No differences were found between the groups in the HADS, but interestingly compared to healthy subjects valve repair and pulmonary autograft patients had significantly higher anxiety scores while mechanical valve replacement patients did not. CAQ scores were comparable between the groups after correction for follow-up, but significantly worse when compared to healthy subjects. Concerning valve-specific questions, mechanical valve replacement patients were more bothered by valve sounds and the frequency of follow-up, and more concerned about failure of the valve and possible bleeding due to anticoagulation medication.

### Thoracic aortic surgery

#### Quality of life after thoracic aortic surgery

Very limited information is available concerning the quality of life after thoracic aortic surgery. A study performed by Olsson in 1999 [21] comparing quality of life in thoracic aortic surgery patients to the general population using the SF-36, showed significantly worse HRQoL outcomes for patients who had thoracic aortic surgery. However, an updated study by Olsson in 2013 [22] using the SF-36 showed comparable results for both groups. This difference could be the result of the advances in cardiac surgery and quality of cardiovascular care throughout the years. Two other studies using the SF-36, as well as the systematic review performed by Jarral et al., found quality of life in thoracic aortic surgery patients to be comparable to the general population [8, 23, 24].

#### Valve sparing aortic root surgery versus composite graft root replacement

Franke et al. [25] investigated the quality of life after valve sparing aortic root surgery and composite graft root replacement using the SF-36. Patients in the replacement group had significantly worse quality of life outcomes in most of the domains of the SF-36 compared to the valve sparing group. In subgroup analyses for age, they found that patients under 50 years of age or over 70 years old had significantly worse outcomes in most of the SF-36 domains in the composite graft group compared to the valve sparing group. Furthermore, composite graft patients were significantly more disturbed by valve sounds, were more afraid their valve would fail and gave a lower score to their overall condition. Jarral et al. [8] reports one study that found no difference in quality of life.

#### Composite graft root replacement: mechanical versus tissue valves

Two studies [26, 27] investigated HRQoL using SF-36, EuroQoL, the Seattle Angina Questionnaire (SAQ), and the Hospital Anxiety and Depression Scale (HADS) in patients after composite graft root replacement, comparing mechanical and tissue valves (Ross procedure and biological valves). Both studies found comparable HRQoL outcomes between the two groups for all instruments used.

### Effect of self-managing anticoagulation

A recent Cochrane review [28] found that patients self-managing their anticoagulation medication had fewer thromboembolic and bleeding complications, with some studies even observing a reduction in mortality. Unfortunately, this review did not address the effect of anticoagulation self-management on HRQoL. Pozzi et al. [29] also performed a systematic review on self-managing anticoagulation therapy, and found eight studies assessing HRQoL with regard to self-managing anticoagulation in both adult and pediatric populations. Seven studies found an improvement in the quality of life and general treatment satisfaction together with lower anxiety, distress and strain for the patient and their family. These observations suggest that in patients who require anticoagulation there is room for the improvement of HRQoL through self-management of anticoagulation therapy, although one should realize that not every patient may be capable of self-management and thus far cost-effectiveness appears comparable to standard anticoagulation management [30].

### The effect of baseline HRQoL on HRQoL outcome scores

When assessing HRQoL after thoracic aortic surgery it is important to take into account HRQoL before the surgery, especially when elective surgery is considered. Kurfirst et al. [31] investigated the effect of preoperative HRQoL scores on HRQoL outcome scores. They found that older patients with lower preoperative HRQoL show a score improvement comparable to younger patients. However, a higher preoperative HRQoL score was a risk factor for non-improvement of postoperative HRQoL scores. In concordance, Koch et al. [32] examined the functional health-related quality of life after aortic valve replacement and found a ‘ceiling effect’ as patients with high preoperative scores could only retain their HRQoL level or worsen. This suggests that patients with high preoperative HRQoL scores, usually the asymptomatic patients, have little to gain and a lot to lose when it comes to HRQoL. This should be discussed with the patient during preoperative consultation and taken into account in treatment decision-making.

### Time factor in studying quality of life after surgery

Another factor that may influence HRQoL after thoracic aortic surgery is time: HRQoL varies with time passing and usually declines with age. Early after surgery HRQoL is usually strongly impaired, as this highly invasive type of surgery has a tremendous impact on both physical and mental quality of life. After the first few months HRQoL will improve and stabilize, and next steadily decline with ageing. In addition, late events related to the surgical implant or to progression of aortic disease may have an impact on HRQoL in a time-related fashion. Very little is known about the time-related variation of HRQoL after thoracic aortic surgery. Among the studies included in this review, only Sedrakyan et al. [16] performed a prospective study, in which they used one point in time after surgery for investigating HRQoL after the baseline results. The retrospective and cross-sectional studies investigating aortic valve surgery generally had a timespan between surgery and investigation of 1.5–2.5 years, with one study investigating HRQoL after 30 years [19]. For aortic surgery the mean time to HRQoL assessment was only 2–4 years. Therefore, it is highly recommendable that prospective studies are initiated comparing baseline HRQoL with outcome HRQoL at different points during follow-up within the same patient population.

### Using the right tools

It is equally important to use the right tools, using different types of questionnaires when appropriate and where necessary. The HADS [33] has been validated to be used during hospital admission. The SF-36 can be used at baseline and during follow-up, as it gives general HRQoL information. In addition, it is widely used and, therefore, generalizable in the cardiovascular domain. Valve-specific or disease-specific questionnaires should be used as well, as the SF-36 lacks aortic valve surgery and thoracic aortic surgery specific issues related to HRQoL.

### Time to treatment equipoise

An interesting new concept in surgical decision making that was recently proposed is Time To Treatment Equipoise (TUTE) [34]. It is a method to compare relative risks of two surgical approaches, for example a high risk surgical procedure (10% early mortality) with a low late mortality hazard (1% per year) versus a low risk minimal invasive procedure (1% early mortality) with a higher late mortality hazard (3% per year). Patients who choose for the first option, are willing to take an early risk to obtain better long term survival, while those who opt for the second option do not take an early risk but have to deal with a worse long-term survival. At some point in time (around 3 years) the survival curves of these two treatment strategies will cross and next diverge. TUTE is defined by the duration of time that elapses after an intervention, before the risk of the intervention is nullified and reversed by the cumulative risk of conservative management. Ultimately, the outcome of interest that should be considered when determining TUTE is not survival but HRQoL adjusted survival, to correct for any differences in HRQoL outcomes between the two surgical strategies. It is expected that expansion of TUTE to also include HRQoL is just a matter of time and will provide patients and doctors with even better tools to weigh treatment decisions.

### QALYs

Another important reason to implement HRQoL measurements in clinical research and clinical care, is their importance in assessing the cost-effectiveness of treatment options. In particular in cardiovascular 21st century healthcare with steadily expanding technological advances in an ageing population, the containment of costs is becoming a holy grail [35]. When balancing risks, benefits and costs of treatment options in cardiothoracic interventions the consideration of HRQoL is essential to be able to calculate quality adjusted life years (QALYs). QALYs are an essential component of cost-utility analysis in order to estimate the cost-per-QALY associated with a health care intervention. This information in turn can be utilized to calculate the incremental cost-effectiveness ratio and assess whether a particular intervention is worth the investment. It is expected that the use of HRQoL data for health economic purposes will become increasingly accepted to guide resource allocation.

## Future directions

### Which tool to use

The SF-36 survey is currently the most popular tool in the field of aortic valve and thoracic aortic surgery. It remains, however, unknown what the effectiveness is of SF36 and the other available HRQoL tools in terms of compliance, validity and sensitivity. It is methodologically challenging to compare scores obtained with different instruments, even if they measure the same health concept. This is due to the dependency of individual items within a survey to come to a sum score. A disadvantage of sum scores is the ‘ceiling effect’, patients with preoperative high HRQoL scores can either remain the same or worsen post-operatively [32]. There are alternative methods originating from educational sciences, where skills can be tested and compared over time taking into account the growing knowledge in primary school children. These methods are based on item response theory (IRT) in combination with a calibrated item bank. In addition, computerized adaptive testing methods can be implemented to keep the burden (number of question asked) to the patient as low as possible. This can be achieved by presenting difficult items to asymptomatic patients and easier items to more severely impaired patients, whilst ensuring that estimates of health status or HRQoL remain completely comparable. The application of this innovative method of measuring is being studied in the ‘Amsterdam linear disability score’ (ALDS) project where functional outcome is measured in several clinical research areas [36]. Despite the complexity of these methods, the use should be explored in the cardiac surgery domain. Furthermore, the nonprofit International Consortium for Health Outcomes Measurement (ICHOM) developed a consensus standard set of outcome measures for coronary artery disease (CAD) and heart failure (HF), where other conditions will follow. This initiative could lead to further standardization [37].

### Practical gaps to overcome

An important limitation in the current use of HRQoL tools in cardiac surgery is its retrospective nature. We need to implement HRQoL in prospective clinical trials, observational cohorts and institutional quality improvement programs to use these measurements prospectively to start continuously improving the quality of care for patients with aortic valve and thoracic aortic disease. A national initiative in the Netherlands termed *‘Meetbaar Beter’* or in English ‘Measurably Better’ encourages participating heart centers to collect HRQoL pre- and post-intervention (at 1 year). The initiative started 5 years ago, and only 3 of the 14 affiliated Dutch heart centers have implemented the HRQoL surveys, exposing the challenges of implementation in clinical practice [38]. One of the reasons why it is not used everywhere and prospectively, is probably due to the fact it is very time consuming—and thus costly—to send questionnaires pre- and post-operatively to the patient, to trace the missing, to build a database and perform data entry. Hopefully in the future implementation will be facilitated by the rapidly evolving information technology possibilities in health care. One example is ‘empowering the patient’ by providing them with secure access to their own electronic medical files, with the ability to fill in HRQoL questionnaires online and store data in institutional secures data ware houses. While the information is stored in the medical file, it can be used in clinical decision making, enabling further self-determination. Moreover, we encourage academic societies to share standalone tools they have developed, to empower research groups worldwide. When the tools are available through an open source, it will catalyze the improvement and usage of these tools through ‘networked science’ [39]. One of the greatest examples is the open source statistical software program ‘R’.

### Disease specific solution for aortic valve regurgitation and thoracic aortic aneurysms

The AVIATOR initiative—under the umbrella of the Heart Valve Society (HVS)—is a start to achieve more standardization in the treatment of patients with thoracic aortic dilatation, aortic valve regurgitation, or both. It is a prospective, multicenter, international registry that has the goal to gather information during the complete trajectory of the disease from diagnosis until long-term follow-up regardless whether the patient needs to be operated upon or not. The database is hosted in Paris and the abilities for patient-self-reported outcomes, is a built-in feature which can be further explored. Patients receive a unique access code by e-mail to fill in questionnaires online. The provided answers will be stored in the database. Participating in AVIATOR is free, provided that one the heart team physicians is member of the HVS. It enables participating centers to implement HRQoL surveys easily for this specific patient group. Within AVIATOR the debate should be encouraged to select the right measurement tool to measure HRQoL. The ultimate goal of AVIATOR is to create an evidence base to enable tailoring the most suitable surgical treatment strategy to individual patients with aortic regurgitation, thoracic aortic dilatation or both.

## Conclusion

In a perfect world, patients would get the best quality of care with regard to outcome measures and quality of life, while still being socially responsible, cost-effective, ethical and with minimum strain on physicians and other healthcare professionals. Practically, this means a patient should receive the best evidence based treatment with the lowest mortality and complication rates, but also the treatment that allows the patient achieve their best HRQoL to live life to the fullest. The lowest complication rates and the best HRQoL unfortunately do not always go hand in hand. Also, the valuation of quantity and quality of life may vary considerably: for example in the growing elderly population the balance between quality of life and quantity of life is very delicate, especially toward the end of life. Our scientific community has the responsibility to fill in these knowledge gaps, providing the physician, the patient, and society with tools to carefully balance decision making.

In light of the (level of) evidence on HRQoL outcomes after aortic valve and thoracic aortic surgery presented above, it is necessary to discuss factors that may be of influence on HRQoL, and to deliberate how the cardiovascular surgery community can proceed to effectively measure what matters to the patient toward the future, and use this knowledge to improve treatment decision-making, patient outcomes, and ultimately optimize health care efficiency.
